# Changes in the distribution of high-risk births associated with changes in contraceptive prevalence

**DOI:** 10.1186/1471-2458-13-S3-S4

**Published:** 2013-09-17

**Authors:** John Stover, John Ross

**Affiliations:** 1 Futures Institute, Glastonbury, CT 06033, USA; 2 Independent Consultant, Wallkill, NY 12589, USA

## Abstract

**Background:**

Several birth characteristics are associated with high mortality risk: very young or old mothers, short birth intervals and high birth order. One justification for family planning programs is the health benefits associated with better spacing and timing of births. This study examines the extent to which the prevalence of these risk factors changes as a country transitions from high to low fertility.

**Methods:**

We use data from 194 national surveys to examine both cross section and within-country variation in these risk factors as they relate to the total fertility rate.

**Results:**

Declines in the total fertility rate are associated with large declines in the proportion of high order births, those to mothers over the age of 34 and those with multiple risk factors; as well as to increasing proportions of first order births. There is little change in the proportion of births with short birth intervals except in sub-Saharan Africa. The use of family planning is strongly associated with fertility declines.

**Conclusions:**

The proportion of second and higher order births with demographic risk factors declines substantially as fertility declines. This creates a potential for reducing child mortality rates. Some of the reduction comes from modifying the birth interval distribution or by bringing maternal age at the time of birth into the ‘safe’ range of 18-35 years, and some comes from the actual elimination of births that would have a high mortality risk (high parity births).

## Introduction

**Figure 1:**
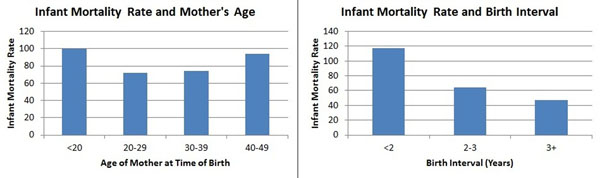
Infant mortality and age of mother (a) and birth interval (b) for developing countries. (Source: Family Planning Can Reduce High Infant Mortality Rates, Guttmacher Institute, April 2002, Issue Brief)

A key point in advocacy for family planning is ‘family planning saves lives’. It is clear that increasing rates of family planning use lead to reductions in fertility and the number of births. The reduction in the number of births leads to fewer maternal deaths, since women are exposed less often to the risks of child bearing. The number of child deaths will also decline because of a smaller number of children exposed to the risk of mortality. Also it has long been recognized that changes in the demographic characteristics of births are associated with changes in child mortality rates [1]. The characteristics most closely associated with child mortality rates are: the age of the mother at the time of birth, the birth interval and the birth order. Data from national household surveys show that child mortality rates are elevated when the age of the mother at the time of the birth is less than 18 or greater than 34, when the interval between one birth and the next is less than 24 months (Figure 1) and when the birth order is greater than 3. These four conditions define births that have a high demographic risk. Previous research has shown that the proportion of high risk births is inversely related to contraceptive prevalence and that the proportion of high risk births is directly related to maternal and child mortality rates [2-5].

Research is ongoing to better understand the causal pathways that lead to this association between contraceptive prevalence and mortality rates. There could be a direct causal relationship arising from maternal depletion or competition among children for resources. Or there could be a relative weak effect mixed with an association that arises because both the proportion of births that are high risk and child mortality rates are related to other factors, such as poverty, access to services or individual characteristics. Other papers in this Supplement explore these topics. This paper focuses on one aspect of this question, the relationship between rising contraceptive use and the proportion of births that are high risk. In other words, as countries transition from very low levels of contraceptive use and high fertility to high levels of contraceptive use and low fertility, how much change do we expect to see in the proportion of births with short birth intervals or high birth order or to mothers who are very young or old?

## Methods and data

We use both cross section comparisons and time series comparisons to examine the relationships between contraceptive prevalence and the distribution of births by risk factor. Although we are ultimately interested in the relationship between contraceptive use and high risk births, contraception is only one factor that affects fertility behavior. The total fertility rate (TFR) is also determined by the proportion of women 15-49 who are in union, the duration of postpartum insusceptibility, sterility and abortion [6,7]. Therefore, we first examine the relationship between the total fertility rate and the distribution of births and then examine the factors that affect the total fertility rate.

Data on the distribution of births by risk factor are available from household surveys conducted by the Demographic and Health Survey (DHS) Project since the 1980s. For this analysis we use data from 194 surveys for 80 countries. The indicators used include the following:

• TFR, total fertility rate, for the three years preceding the survey

• CPR, contraceptive prevalence, percentage of currently married women currently using any form of contraception including traditional methods, and by method used

• Marriage, percentage of women 15-49 currently married or living together

• PPI, median duration (in months) of postpartum insusceptibility following births in the three years preceding the survey. Postpartum insusceptibility is the period after a birth when a woman is not exposed to the risk of pregnancy because of postpartum abstinence or postpartum amenorrhea.

• Birth distribution, the percentage of children born in the five years preceding the survey by risk factor

○ Not in any high risk category.

○ First birth to mothers 18-34 years of age.

○ Mother’s age < 18 years.

○ Mother’s age > 34 years.

○ Birth interval < 24 months.

○ Birth order > 3.

○ In any avoidable high risk category.

It would be ideal if we had data from a large number of countries showing the change in the distribution of births by risk factor as fertility declines from high levels to low levels. Unfortunately, the most complete source of information on this topic, the Demographic and Health Surveys, did not start early enough to capture the entire fertility transition for countries that currently have low fertility. Using cross sectional data we can explore the entire range from TFR of 7 down to 2, but the results can be affected by regional differences in fertility as well as trends within countries. We partially address this issue by including multiple surveys from the same country in the cross national data set. We also present data on changes in TFR and changes in the distribution of births within countries to reinforce the conclusions from the cross section analysis.

## Results

### High risk births and TFR

#### Cross sectional analysis

Across all 194 surveys, there is considerable variation in the distribution of births by risk factor as shown in Table 1.

**Table 1:** Distribution of births by risk factor across 194 national household surveys

Risk Factor	Mean	Median	Range
Not in any risk category	25%	25%	9-43%
First birth to mothers age 18-34	19%	17%	8-52%
Mother’s age < 18	6%	6%	0.5-17%
Mother’s age > 34	1%	1%	0.1-6%
Birth interval < 24	8%	7%	3-19%
Birth order > 3	20%	21%	1-33%
Mother’s age <18 and birth interval < 24 months	0.6%	0.5%	0-2.2%
Mother’s age > 34 and birth interval < 24 months	0.1%	0.1%	0-0.6%
Mother’s age > 34 and birth order > 3	10%	10%	1-18%
Mother’s age > 34 and birth interval < 24 months and birth order > 3	2%	2%	0-6.5%
Birth interval < 24 months and birth order > 3	7%	6%	1-20%

**Figure 2:**
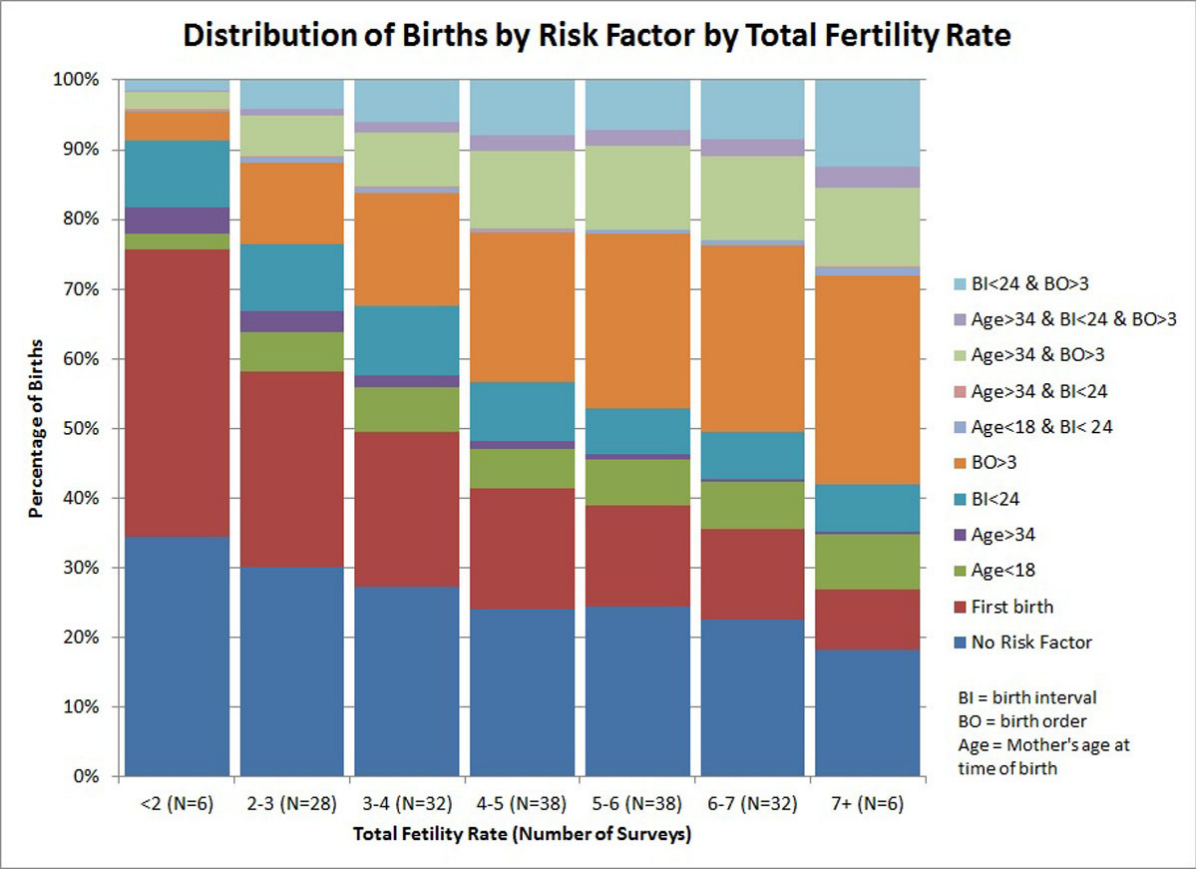
Distribution of Births by Risk Factor and TFR

The distribution of births varies by level of TFR. Figure 2 shows the average percentage of births by risk factor for all the surveys with TFR in the corresponding category. At the highest levels of TFR (6+) 25%-35% have either no risk factors or are first births (considered unavoidable) and about 25% have more than one risk factor. At the lowest levels of TFR over 58% of births are first births or have no risk factor and less than 12% have multiple risk factors. The most striking changes are the increase in first births (from 9% at TFR 7+ to 40% at TFR <2) and the decrease in high order births (from 30% at TFR 7+ to 4% at TFR < 2).

**Figure 3:**
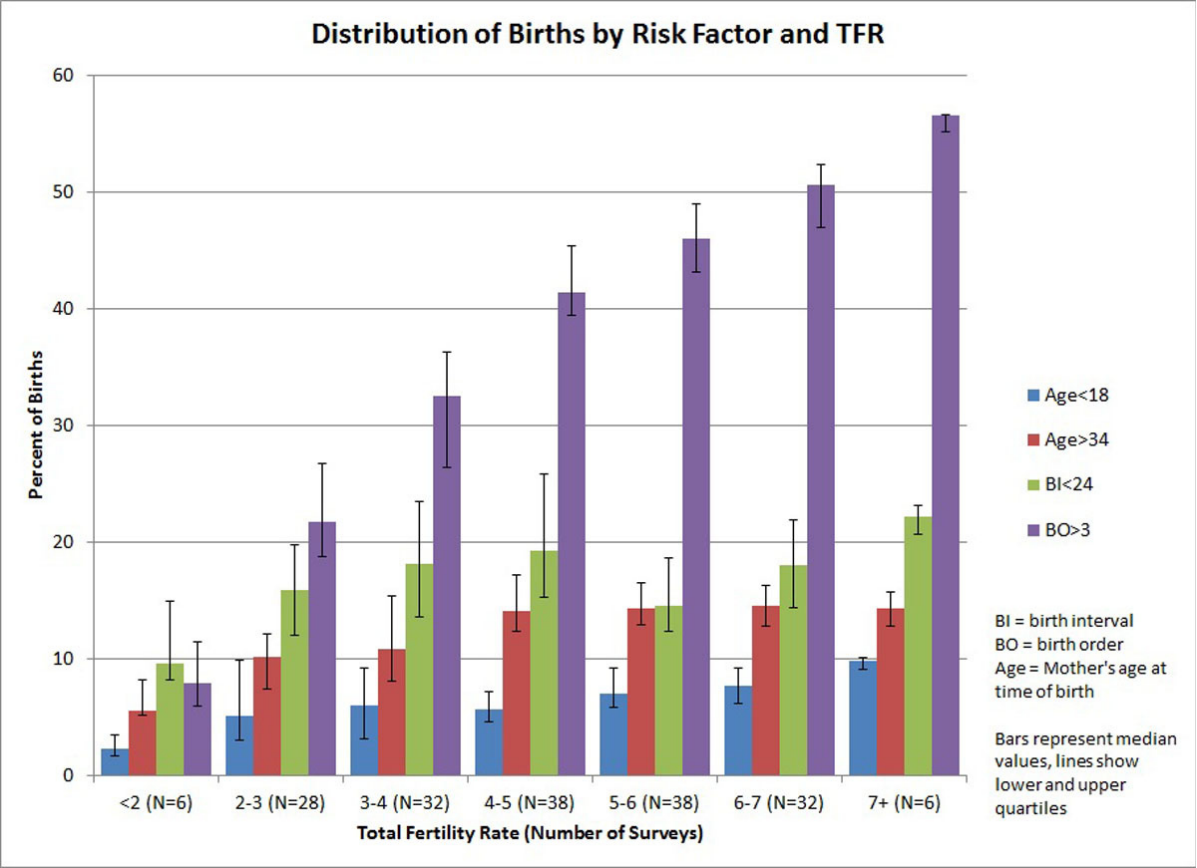
Distribution of Births by Single Avoidable Risk Factor versus TFR

Figure 3 shows the distribution of births among the single avoidable risk factors (not including first births which cannot be avoided). (In this chart a single birth can be in more than one category if it has multiple risk factors.) At high levels of TFR the major risk factor is high birth order, accounting for more than half of all births. At low levels of TFR no single risk factor accounts for more than 10% of births. At most TFR levels high birth order dominates.

**Figure 4:**
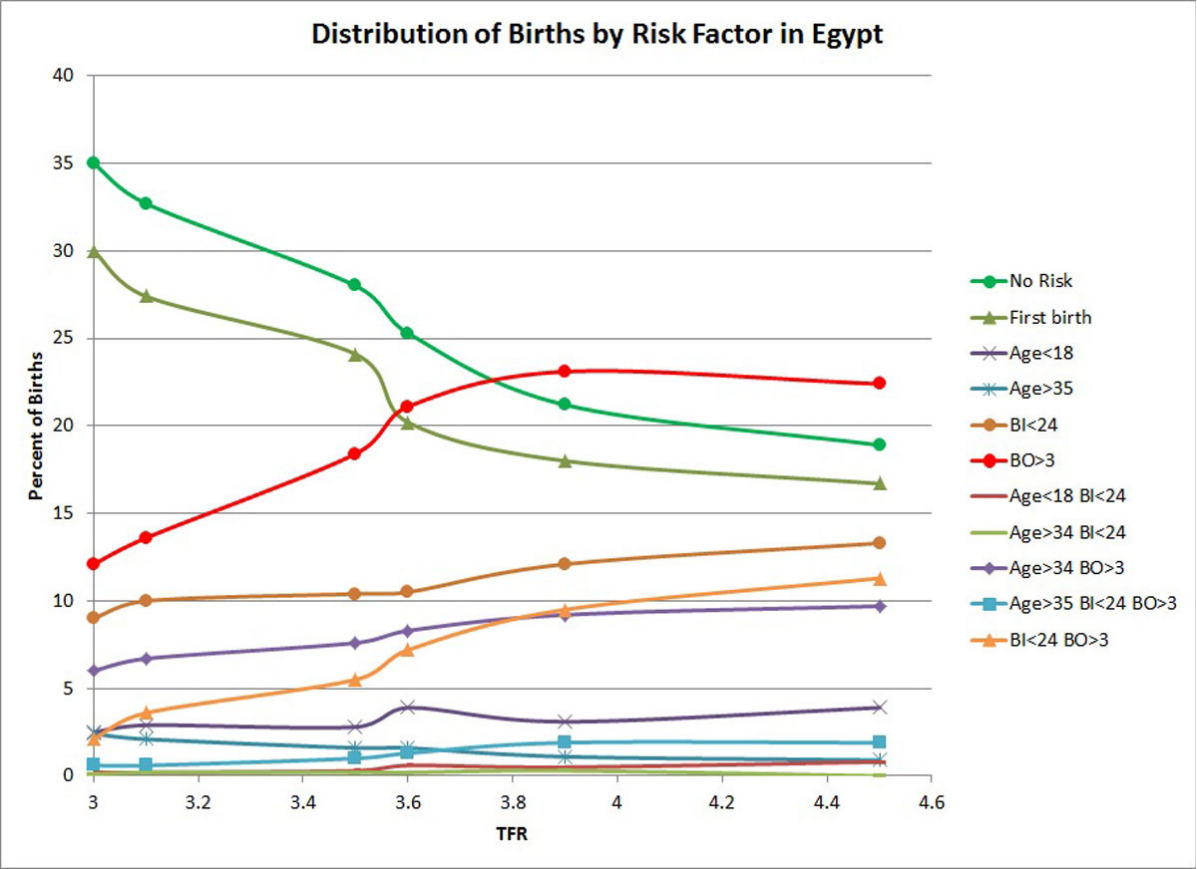
Distribution of births by risk factor in Egypt

Figures 2 and 3 combine cross section and time series data. Some countries are represented by as many as 6 surveys while others contribute only one. As a result the relationships shown are affected by the fact that data for the highest fertility categories are primarily from countries in sub-Saharan Africa while data for the lowest fertility levels are primarily from Asia and Latin America. However, for countries with many surveys covering a wide span of TFRs similar patterns appear. Figure 4 shows the distribution of births by risk category for Egypt across six surveys as TFR fell from 4.5 to 3.0. It shows a strong rise in the proportion of births with no risk and first order births as TFR falls, and a decreasing proportion of births with high birth order.

**Figure 5:**
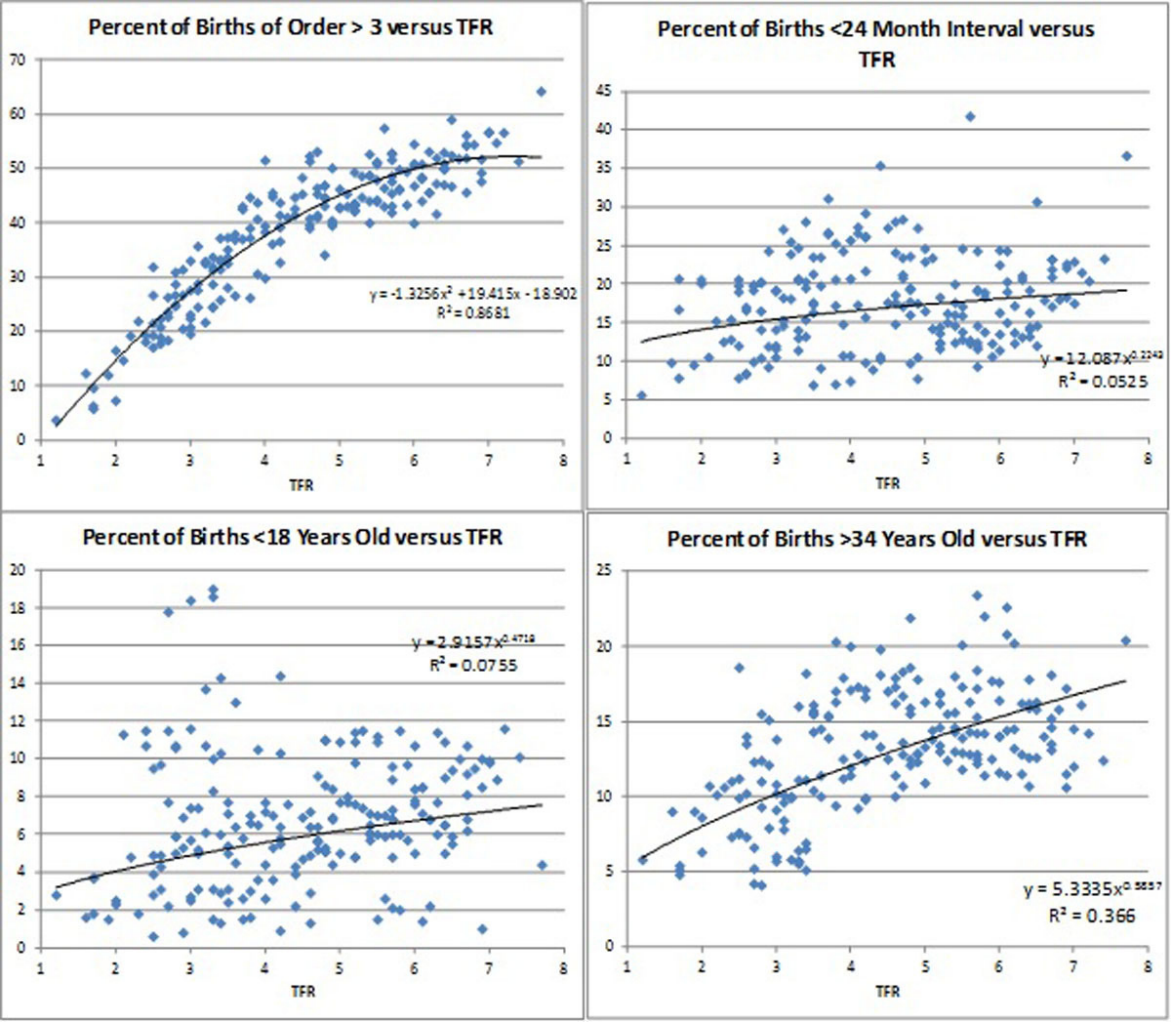
Cross sectional correlations between the percentage of births with risk factors and TFR

Figure 5 shows cross plots for the four individual risk factors shown in Table 1 versus TFR. The correlation with TFR is highest for the percentage of births with high birth order (R2 = 0.87) which varies from over 50% at high levels of TFR to under 10% at low levels. The correlation with TFR for mothers’ age is weaker; the percentage of births occurring to mothers over age 34 varies from nearly 20% at high levels of TFR to 5% at low levels of TFR. The association is much weaker for birth interval and mother’s age below 18. The association with TFR is generally strong for categories with multiple risk factors and more than 1 percent of all births: 0.76 with mother’s age over 34 and birth order greater than 3; 0.56 for birth interval less than 24 months and birth order greater than 3; and 0.61 for mother’s age greater than 34, birth interval less than 24 months and birth order greater than 3.

The relationship between CPR and risky births is similar to that with TFR but weaker. The correlation with high order births is 0.68 versus 0.87 with TFR; for mother’s age over 34 it is 0.28 compared to 0.37 with TFR. The correlations between CPR and birth interval and young age at birth are similarly low, 0.05 and 0.06.

These cross section comparisons with TFR might be misleading since the surveys with the lowest levels of TFR are all from countries in Eastern Europe, Asia and South America while those with the highest levels of TFR are mostly from Sub-Saharan Africa. For Sub-Saharan Africa alone, the correlation with high order births remains strong at 0.73 compared to 0.87 for all surveys. Notably, the correlation with birth interval increases to 0.45 for Sub-Saharan Africa compared to just 0.05 for all countries. For mother’s age the correlations are only 0.01 for age less than 18 and 0.001 for age greater than 34.

#### Within country analysis

**Figure 6:**
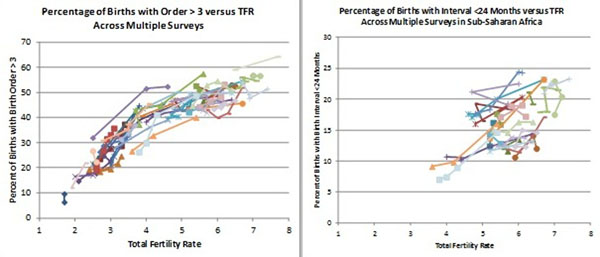
Changes in the percentage of births with risk factors versus TFR in each country

The above cross section comparisons combine differences between countries with differences between surveys for the same country. Since most of the countries in this data set have more than one survey we can also compare changes in the distribution of births with changes in TFR. Figure 6 on the left shows the changes in the percentage of births with high birth order with changes in TFR for all countries with multiple surveys (each line shows the movement of the two measures through time across surveys in the same country). The pattern is similar to the cross section patterns shown earlier. The association between TFR and birth interval is weak when all countries are included, but there is an association for the countries in Sub-Saharan Africa as shown in the right hand chart in Figure 6. No pattern is evident when comparing changes in the percentage of births to mothers below the age of 18 or above the age of 34 with changes in TFR.

### Factors affecting the total fertility rate

The fertility rate is affected by a number of factors including contraceptive use, the proportion of women who are sexually active, postpartum infecundability, sterility and abortion. Here we examine each of these factors.

#### Contraceptive use

There is a strong correlation between contraceptive prevalence and TFR. For this data set the R2 for a linear trend is 0.73. The correlation is considerably weaker (0.60) when contraceptive prevalence is limited to modern methods only, indicating that traditional methods do play a role in regulating fertility.

**Figure 7:**
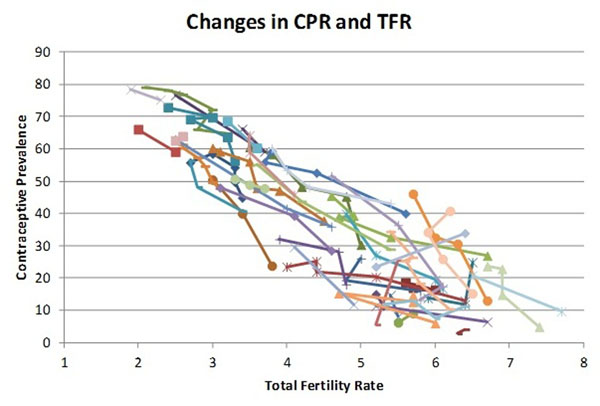
Changes over time in contraceptive prevalence between successive surveys and changes in the total fertility rate in each country

For this analysis we are concerned more with changes than absolute levels so we also compare changes in contraceptive prevalence with changes in TFR. Figure 7 compares these changes for 44 countries with multiple surveys, again with one line per country. The pattern of changes is similar to the cross section comparison. Overall the average change is a decrease in TFR by one birth per woman for every increase in contraceptive prevalence of about 17 percentage points.

#### Additional factors

Over the course of the fertility transition as TFR drops from 7-8 to 2 or less, contraceptive prevalence increases from less than 10% to almost 80% in the cross sectional analysis. Changes in the other proximate determinants of fertility are much smaller. The median duration of postpartum insusceptibility drops from 15 months for surveys with TFR 6 and above to about 5 months at the lowest levels of TFR. This change would lead to shorter birth intervals and higher TFR if it were not associated with increasing levels of contraceptive use. The mean age at marriage rises from about 16 at high levels of TFR to about 20 at lower levels. This change alone would lead to somewhat lower fertility but the effect is not significant when the effects of CPR are also included.

Changes in abortion rates can have a significant effect on TFR. For countries with data the total abortion rate (TAR, abortions per woman during her lifetime) drops from 3-4 at low levels of *modern* contraceptive use to nearly zero at very high levels (as contraception replaces abortion for limiting the number of births) but has the opposite relationship to the prevalence of *traditional* methods of family planning (as abortion is used in the case of method failure) [8]. The proximate determinants of fertility model estimates that at low levels of contraceptive use each abortion averts about 0.4 births (less than one birth because of the rapid return to fecundity). The impact can be larger at higher levels of contraceptive use.

Across all 194 surveys there is considerable variation in risk ratios for child mortality but the data confirm that these demographic factors are associated with higher risk of child mortality as shown in Table 2. The figures shown in this table represent the ratios of the proportion of births in the last five years that have died in each risk category to the proportion dead among births not in any risk category. The median risk ratio is the median value across all 194 surveys, while the lower and upper quartiles show the spread around the median for these surveys. The highest risk ratio (2.9) occurs for children with a combination of three risk factors (mother older than 34, birth interval less than 24 months and birth order greater than 3). The second highest (2.8) is for children with short birth intervals and mothers under the age of 18. The highest risk (1.8) for children with a single risk factor occurs those with a mother under the age of 18. Some possible categories are excluded if the proportions of births in those categories are quite small, for example high birth order births occurring to mothers under the age of 18.

**Table 2:** Risk ratios for child mortality by birth characteristic

Risk Factor	Median Risk Ratio	Lower Quartile	Upper Quartile
Not in any risk category	1	1	1
First birth to mothers age 18-34	1.2	1.0	1.4
Mother’s age < 18	1.8	1.4	2.0
Mother’s age > 34	1.2	0.6	1.6
Birth interval < 24	1.6	1.3	1.9
Birth order > 3	1.2	1.0	1.4
Mother’s age <18 and birth interval < 24 months	2.8	1.4	3.2
Mother’s age > 34 and birth interval < 24 months	1.8	2.0	2.8
Mother’s age > 34 and birth order > 3	1.4	1.0	1.6
Mother’s age > 34 and birth interval < 24 months and birth order > 3	2.9	1.8	3.5
Birth interval < 24 months and birth order > 3	2.3	1.7	2.7

## Discussion

The distribution of high risk births changes dramatically as countries transition from high fertility to low fertility. The most striking changes are a reduction in births with multiple risk factors and those with high parity. This is a natural part of the transition as average number of births per woman cannot decline significantly without a reduction in the proportion of high order births.

Changes in the proportion of births at maternal age 35 and above are also associated with declines in the TFR as women complete their child bearing at younger ages. There is little change in the proportion of births to mothers below the age of 18 as TFR declines. Across all countries there is not much change in the percentage of births with short birth intervals either, but this does occur in Sub-Saharan Africa. That may reflect the greater focus in the region on birth spacing as a rationale for family planning.

There is a strong correlation between contraceptive prevalence and TFR. Thus, as CPR rises TFR falls and the distribution of births by risk factor changes. CPR is the major factor associated with changes in TFR. Changes in the proportion married and post partum insusceptibility are much smaller and less directly associated with TFR changes. Changes in abortion rates may also affect TFR but the data are not good enough to determine the exact relationship. One would expect that abortion would reduce the proportion of births with short intervals and reduce the number of high parity births but the degree of the effect is not clear.

It is clear from these data that as contraceptive prevalence increases TFR falls and a smaller percentage of births have one or more of these four risk factors that are associated with high child mortality. This creates a potential for reducing child mortality rates. Some of the reduction comes from modifying the birth interval distribution or by bringing maternal age at the time of birth into the ‘safe’ range of 18-35 years, and some comes from the actual elimination of births that would have a high mortality risk (high parity births).

This analysis does not explain the mechanisms by which these risk factors lead to higher mortality rates. But whatever the mechanisms are it seems evident that the expansion of the use of family planning is key to achieving a healthier distribution of births.

## Competing interests

The authors declare that they have no competing interests.

## Authors’ contributions

JS and JR jointly conceived the paper and conducted the analysis. JS wrote the first draft of the paper. JS and JR reviewed and approved the final draft.

## Declarations

The publication costs for this supplement were funded by a grant from the Bill & Melinda Gates Foundation to the US Fund for UNICEF (grant 43386 to "Promote evidence-based decision making in designing maternal, neonatal, and child health interventions in low- and middle-income countries”). The Supplement Editor is the principal investigator and lead in the development of the Lives Saved Tool (LiST), supported by grant 43386. He declares that he has no competing interests.

This article has been published as part of *BMC Public Health* Volume 13 Supplement 3, 2013: The Lives Saved Tool in 2013: new capabilities and applications. The full contents of the supplement are available online at http://www.biomedcentral.com/bmcpublichealth/supplements/13/S3.
